# Protective Efficacy of BCG Overexpressing an L,D-Transpeptidase against *M. tuberculosis* Infection

**DOI:** 10.1371/journal.pone.0013773

**Published:** 2010-10-29

**Authors:** Scott T. Nolan, Gyanu Lamichhane

**Affiliations:** Department of Medicine, Johns Hopkins University School of Medicine, Baltimore, Maryland, United States of America; Texas A&M University, United States of America

## Abstract

**Background:**

*M. bovis* Bacille Calmette-Guérin (BCG), currently the only available vaccine against tuberculosis (TB), fails to adequately protect individuals from active and latent TB infection. New vaccines are desperately needed to decrease the worldwide burden of TB.

**Methods and Findings:**

We created a recombinant strain of BCG that overproduces an L,D-transpeptidase in order to alter the bacterial peptidoglycan layer and consequently increase the ability of this immunogen to protect against virulent *M. tuberculosis* (*Mtb*). We demonstrate that this novel recombinant BCG protects mice against virulent *Mtb* at least as well as control BCG, as measured by its ability to reduce bacterial burden in lungs and spleen, reduce lung histopathology, and prolong survival. A nutrient starved recombinant BCG preparation, while offering comparable protection, elicited a response characterized by elevated levels of select Th1 cytokines.

**Conclusions:**

Recombinant BCG overexpressing a L,D-transpeptidase that is nutrient starved elicits a stronger Th1 type response and is at least as protective as parent BCG. Results from this study suggest that nutrient starvation treatment of live BCG vaccines should be further investigated as a way to increase host induction of Th-1 related cytokines in the development of experimental anti-TB vaccines.

## Introduction

Tuberculosis (TB) still remains a major global health threat as more lives were expected to be lost in 2009 than any previous year in history [1]. In 2008, the latest year for which data is available from the World Health Organization, there were ∼9.4 million incident cases of active TB around the world with 13–16% of these patients being co-infected with HIV [2]. A major reason for the high rate of active TB is reactivation from the vast reservoir of latent infection into active disease. Latent TB describes a person that harbors *M. tuberculosis* (*Mtb*) bacilli, the etiological pathogen of TB, but is clinically asymptomatic [3]. An estimated 2 billion humans are latently infected with *Mtb* [4], each with a 10% likelihood of developing active TB during their lifetime [5]. Worse, those patients co-morbid with HIV have been shown to carry an increased risk of 10% likelihood of reactivation into active TB in a year [6], [7].

It is widely known that the intervention with the highest impact on prevention of TB is a vaccine that can eliminate latent *Mtb* infection. Although protective against childhood forms of disseminated TB and TB meningitis, BCG vaccines are ineffective against latent *Mtb* infection. One widely held view is that a vaccine that more closely mimics the physiology of *Mtb* during latent infection will elicit an effective immune response against these bacilli and consequently may promote elimination of the infection. An understanding of the physiological state of the *Mtb* during latent infection is a prerequisite in order to identify antigens specific to this state for development of a vaccine that can elicit protection to prevent latent infection.

*In vitro* models designed to mimic the physiology of *Mtb* in the dormant state are based on hypoxia, nutrient starvation, and exposure to acid pH and nitric oxide as these conditions are speculated to prevail in the lesion of latent infection [8], [9], [10], [11], [12]. A major drawback of this approach is that *in vitro* conditions often mimic only a single aspect of a complex *in vivo* environment. Two technical hurdles in studying latent *Mtb* infection *in vivo* are the low number of bacilli, which is the quintessential aspect of this infection, and the inability to reliably isolate these bacilli from lungs of a host while preserving their physiological state. Therefore, we have developed a nutrient starvation model to isolate live vaccine bacilli in a dormant state and assess its ability to protect the host against latent TB.

The molecular changes that govern the dormant state of *Mtb* have yet to be fully characterized, but antigens specific to this state have been reported [13], [14] which have recently been shown to induce immune responses specific to latent infection in humans [15], [16]. Expressing antigens associated with dormancy in a recombinant BCG vaccine is a novel and promising strategy, which we have considered in our study. It was shown that *Mtb* remodels its peptidoglycan layer to predominantly contain 3→3 transpeptide cross linkages when transitioning into the stationary phase of growth *in vitro* [17]. We showed previously that expression of an L,D-transpeptidase, Ldt_Mt2_, which alters peptidoglycan cross linkages, is increased during stationary phase, and is required for survival during the chronic stage of growth in mice [18]. In an excellent study, Clarke et al. show that murein fragments from the peptidoglycan layer of bacteria prime the host immune system for response against the pathogens through the Nod1 signaling pathway [19]. These novel observations have led us to hypothesize that overproduction of Ldt_Mt2_ in *M. bovis* BCG may render it selectively more immunogenic against dormant bacilli in an *Mtb* infection, specifically during the chronic phase, as this strain could furnish murein that mimics the altered composition of peptidoglycan layer in dormant *Mtb*.

We have created a recombinant strain of *M. bovis* BCG to express an *Mtb* L,D-transpeptidase and profiled the immune response elicited by this strain and the protection provided by it against virulent *Mtb* using the mouse model of TB.

Although the mouse is not the perfect model of human TB, as its granulomas do not caseate and cavitate (which are hallmarks of the pathology of TB in humans), the mouse is the most widely used model for vaccine studies [20], and it is an effective model of *Mtb* growth in the lung and the mortality associated with it. As is commonplace during the first phase of preclinical evaluation of candidate anti-TB vaccines [20], this study was designed to detect vaccine-attributable CFU reductions in the lungs of immunized mice, as well as to determine the ability of the immunogen to prolong life and protect the lungs from pathological signs of disease. Equally important in the design of this study, is the characterization of the host immune response both to the immunizing agent, and subsequent virulent challenge. Over the past decade, immunologists have detected striking similarities between the human and mouse immune systems resulting in the commercial production of an array of inexpensive, high quality reagents and assays designed to detail the immune response [21] - data which is important in assessing the immunogenic potential of an anti-TB vaccine candidate [22] but is difficult to obtain and expensive, if available at all for larger animal models.

## Results

### Recombinant BCG strain overexpressing Ldt_Mt2_

We created a recombinant BCG strain to overexpress *Ldt_Mt2_ (Rv2518c)*. For this we used *M. bovis BCG* Copenhagen (Danish strain 1331, BCG) as the host strain and transformed it with pGS400H-2518, a Gateway destination plasmid derived from pMV361 [23] into which *ldt_Mt2_* was cloned for expression from the *hsp60* promoter (Fig. S1). Although the host strain possesses an orthologue of *ldt_Mt2_* we hypothesize, based on previous studies, that an increase in the expression of this enzyme from an exogenous copy of the gene will result in remodeling of the peptidoglycan layer with extensive 3→3 transpeptide linkages that is characteristic of *Mtb* in stationary phase [17] and thereby mimic the constitution of peptidoglycan during latency. Peptidoglycan fragments from bacteria are highly immunogenic and are recognized by pattern recognition protein Nod1 and 2 [19]. The genotypes of BCG transformants were verified by Southern blotting: two of the tested recombinant strains were of the desired genotype carrying a single copy of the plasmid at the *attB* site in the bacterial chromosome (Fig. S2). Next, we isolated total RNA from the recombinant BCG and the parent strain to verify the levels of expression of *ldt_Mt2_*. This analysis revealed that *ldt_Mt2_* was expressed at ∼58 fold higher levels in the recombinant strain relative to the parent BCG strain. It is worth mentioning that we could detect no *in vitro* growth defects in recombinant BCG (data not shown). This strain is referred to as rBCG hereafter in the text.

### *In vivo* growth of vaccine strains

**Figure 1:**
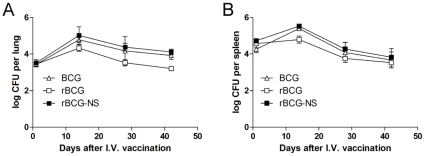
*In-vivo* growth of immunogens. Growth of control BCG (triangle), rBCG (squares), and rBCG that has been nutrient starved *in vitro* (rBCG-NS) (filled squares) in the lung (A) and the spleen (B) of C57/BL6 mice one day and 2, 4, and 6 weeks following immunization. Total CFU counts from 3 mice per data point were log transformed and errors represent standard deviations of log transformed CFU counts.

We administered approximately 6 log_10_ CFU of BCG, rBCG, or rBCG-NS intravenously to mice. As shown in Fig. 1, there was minor variability in the numbers of implanted bacilli, as determined by day +1 CFU counts from the spleen (Fig. 1B). It is worth noting that after one day after immunization, there was even less variation in number of bacilli recovered in the lung (Fig. 1A). These data provided evidence that after culture *in vitro*, rBCG delivered while in log phase or after nutrient starvation exhibit no dissemination defects compared with the parental strain. It has been shown that live BCG vaccine must replicate in the host to be effective [24], therefore, we assessed the natural growth of the vaccine strains over time before infecting the host with virulent Mtb. The lungs and spleens of mice at predetermined times before challenge were homogenized and diluted onto commercial 7H11 Selective plates to recover viable bacilli. All immunogens showed similar *in vivo* growth in both the lung and the spleen (Fig. 1), and exhibited similar *in vivo* growth patterns observed in comparable studies [25].

### Protective effect and immunological response elicited by rBCG

**Figure 2:**
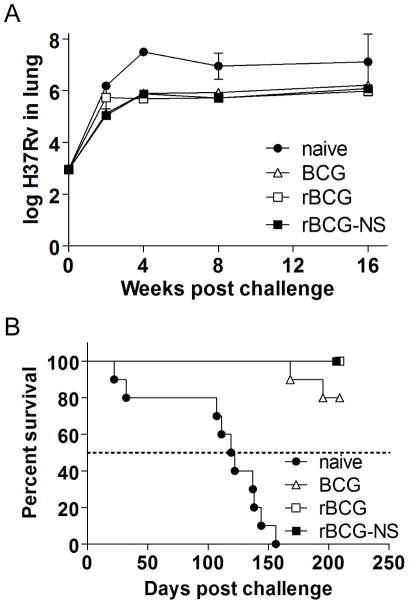
Assessment of protection in immunized mice. (**A**) Growth of virulent H_37_Rv in the lungs of mice that were immunized with either control BCG (triangles), rBCG (squares), and rBCG that has been nutrient starved *in vitro* (rBCG-NS) (filled squares) as assessed 1 day and 2, 4, 8, and 16 weeks after challenge. (**B**) Kaplan-Meier survival analysis of naïve and immunized mice after challenge with a high dose of *Mtb*.

We evaluated the protective efficacy of rBCG by assessing the following: (a) the extent to which it limited growth of virulent strain of *Mtb* in the lungs and spleen of mice, (b) the extent of morbidity based on histopathological observations of disease in the lungs, and (c) the additional time (days) mice survived as a result of receiving rBCG. In addition, we studied the immune response elicited by rBCG by profiling 7 different TB-specific cytokines and chemokines at different stages of immunization and subsequent challenge. A hallmark of the BCG vaccine is its ability to elicit an immune response which limits the growth of virulent *Mtb* and halts the progression to active disease. At four weeks following challenge, the time during which bacterial load peaks, the burden of *Mtb* in the lungs (expressed in mean log_10_ bacilli per organ) was 7.50±0.12, 5.89±0.17, and 5.69±0.09 in the lungs of naïve, BCG and rBCG immunized mice respectively (Fig. 2A). A similar trend in CFU reductions compared to naïve mice was observed at 8 and 16 weeks following challenge. This data demonstrated that our experimental rBCG elicited an immune response that limited the growth of *Mtb* in the lungs during all phases of infection at a statistically significant level (p<0.001).

In a separate experiment to assess the ability of the immunizing strain to prolong time to mortality following a high dose challenge with the virulent strain, we observed 100% mortality of naïve mice with median survival time of 120.5 days. In contrast, there were no deaths or signs of mortality in mice immunized with rBCG through 209 days after infection, at which time the experiment was terminated, as per our design (Fig. 2B).

**Figure 3:**
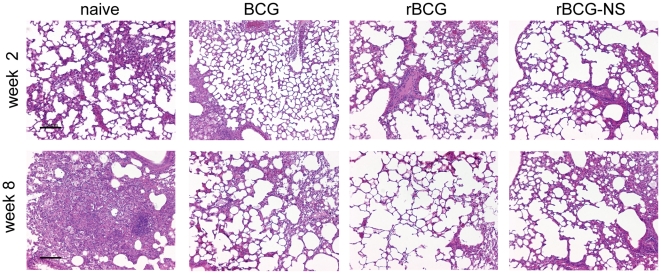
Histopathology of lung tissue after challenge. Sectioned lung tissue from naïve and immunized mice taken 2 and 8 weeks after virulent challenge (all panels 100x magnification, H&E stained).

The decrease in bacterial burden in mice that received rBCG correlated with a decrease in lung consolidation and number of pathological lesions when compared to the group that did not receive any immunization (Fig. 3). Histopathological observations of lungs from all mice at two weeks post challenge revealed healthy lung parenchyma. This was expected as recruitment of T-lymphocytes and consequent cellular infiltration peaks after 2–3 weeks of infection in mice [26]. However, after eight weeks of infection, cellular infiltration and consolidation of the lungs was observed in naïve mice but was absent in lungs of rBCG immunized and control mice.

**Figure 4:**
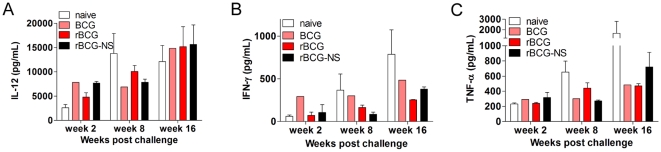
Th1 cytokine induction post-challenge. Total levels (in pg/mL) of Th1 cytokines (**A**) IL-12, (**B**) IFN-γ and (**C**) TNF-α measured in the lungs of naïve, BCG, rBCG, or rBCG-NS immunized mice one day, and 6 weeks following immunization.

Activation of the T-helper type 1 (Th1) response resulting in robust cell mediated immunity plays a dominant role in combating intracellular pathogens such as *Mtb* [26]. IL-12 has been shown to direct the adaptive immune response to a Th1 type [27]. Two weeks following challenge, the levels of IL-12 in the lungs of naïve and rBCG immunized mice were 2606 pg/mL and 4797 pg/mL respectively, however levels of this cytokine in these two groups of mice were not different at later time points (Fig. 4A). IFN-γ, the major effector cytokine of the Th1 pathway, has been shown to be critical to mounting an effective immune response [28], [29]. Although IFN-γ levels were similar during the acute stage following challenge with *Mtb,* we observed significantly diminished levels of IFN-γ in rBCG immunized mice during the chronic stage of infection compared to naïve mice (Fig. 4B). TNF-α is known to act in concert with IFN-γ to initiate production of reactive nitrogen molecules resulting in an enhanced killing of *Mtb* within macrophages [30]. Two weeks after challenge, we detected similar levels of TNF-α in the lungs of naïve and rBCG-immunized mice. However, during the chronic period of infection, TNF-α levels were approximately 2-fold higher in the naïve mice possibly reflecting an attempt by the host to limit proliferation of *Mtb* (Fig. 4C).

### Effects of *in vitro* nutrient starvation on protection elicited by rBCG

One of our hypotheses is that bacilli isolated in a dormant state from nutrient starved cultures mimic bacillary physiology during latent infection and therefore can elicit an immune response that is selective against latent TB. To test this hypothesis, mice were immunized with a preparation of nutrient starved rBCG (referred to as rBCG-NS hereafter), and its protective efficacy was assessed by challenging mice with a virulent strain of *Mtb.* During the initial phase of infection mice immunized with rBCG-NS inhibited growth of virulent *Mtb* robustly. Considerably less CFU (0.69 log_10_ difference) of the virulent strain was observed in the lungs of rBCG-NS immunized mice after 2 weeks of infection compared to the control group of mice that received rBCG obtained at the log phase of growth. Four weeks following challenge with virulent *Mtb*, the burden of this strain in the lungs (expressed in log_10_ bacilli per organ) was 7.50±0.12, 5.69±0.09, and 5.88±0.12 per lung of naïve, rBCG and rBCG-NS immunized mice respectively (Fig. 2A). On and after 4 weeks of infection slight differences in mean bacterial burden of the challenge strain were detected in rBCG-NS and rBCG immunized, however these differences were not statistically significant. We assessed lung histopathology in mice immunized with rBCG-NS and observed less lung consolidation and cellular infiltration compared to non-immunized control mice. However, we were unable to detect qualitative differences in the pathology from lungs of mice immunized with either rBCG-NS or rBCG (Fig. 3).

We also evaluated the ability for rBCG-NS to elicit an immune response to protect against death from an aerosol infection intended to cause acute mortality. A time-to-death comparison between mice immunized with nutrient starved and exponentially grown rBCG revealed that although mice that received either immunogen were significantly protected against death compared to naïve controls, there were no deaths or morbidity in mice that were immunized with either preparation of rBCG after 209 days of infection when the experiment was terminated, as per our design (Fig. 2B).

**Figure 5:**
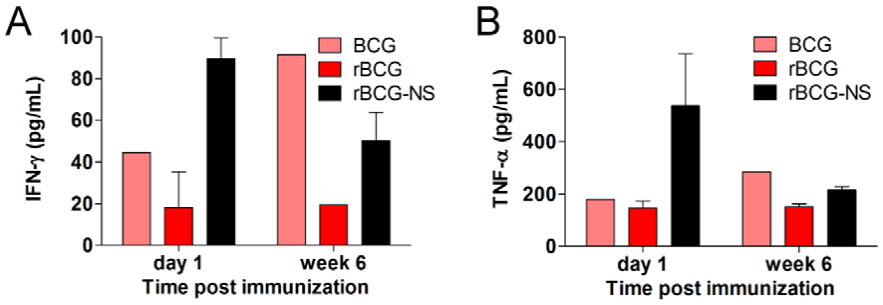
Th1 cytokine induction pre-challenge. Total levels (in pg/mL) of Th1 cytokines (**A**) IFN-γ and (**B**) TNF-α measured in the lungs of BCG, rBCG, or rBCG-NS immunized mice one day, and 6 weeks following immunization.

In order to quantify the measurable aspects of the immune response in immunized animals, total levels of cytokines were determined in the lungs of mice that received rBCG-NS. This assessment revealed differences in the immune response elicited by the experimental vaccines: approximately 60% more of the Th1 response inducing cytokine IL-12 was detected in the lungs of mice immunized with rBCG-NS when assessed 2 weeks post challenge, compared to mice that had received rBCG (Fig. 4A). At this stage, we detected 7,666 pg and 4,797 pg of IL-12 in the lungs of mice immunized with rBCG-NS, and rBCG respectively. Additionally, higher levels of IFN-γ were detected in the lungs of mice immunized with rBCG-NS when assessed before challenge. One day following immunization, we detected 89.65 pg and 18.17 pg of IFN-γ in the lungs of rBCG-NS and rBCG immunized mice respectively (Fig. 5A). Similarly, just prior to challenge (6 weeks post immunization) we detected 50.2 pg and 19.5 pg of IFN-γ in the lungs of rBCG-NS and rBCG immunized mice respectively. Higher levels of TNF-α were detected one day following immunization in the lungs of mice that received rBCG-NS: we measured 537.8 pg and 146.4 pg of TNF-α in the lungs of rBCG-NS and rBCG immunized mice respectively (Fig. 5B). An increase in these Th1 cytokines provides evidence that mice immunized with a nutrient starved preparation of rBCG were able to induce a more pronounced immune response compared with mice that were immunized with rBCG prepared from a culture at log phase of growth.

## Discussion

Several live attenuated vaccines have been engineered in an attempt to reduce the worldwide TB burden. Efficacy of these experimental vaccines has been evaluated preclinically [31], [32], [33], [34], [35], or most recently in humans [36], [37]. To our knowledge only one vaccine candidate, AERAS-407, is designed to target latent TB [38]. An ideal vaccine against latent TB would elicit an immune response that specifically targets *Mtb* bacilli in all organs and tissues during latent infection, and consequently would induce the reduction or elimination of these bacilli. The major reasons for a lack of a vaccine against latent TB are (a) very little is known about the physiology and the antigenic profile of *Mtb* during latent infection, and (b) the lack of a reliable model of human TB latency. *In vitro* models that mimic a singular aspect of bacillary physiology during latency have been developed and these include nutrient starvation [10], oxygen depletion [9], nitric oxide stress [11] and acid stress [8], [12]. In this study we chose to use nutrient starvation as the stress to approximate the physiology of *M. tuberculosis* during latency.

We re-engineered BCG, the only vaccine currently in use against TB, to over produce the transpeptidase Ldt_Mt2_ in an attempt to create a strain whose peptidoglycan layer would closely mimic that of bacilli during dormancy. It was recently reported that expression of Ldt_Mt2_ is elevated during the non-replicating stationary phase [18]. We hypothesized that this recombinant BCG overexpressing *ldt_Mt2_* isolated after nutrient starvation stress would illicit an immune response to specifically target bacilli in a latent infection.

We evaluated the recombinant BCG constructed in this study in mice and found that it protected mice at least as well as control BCG in all measurements of protection performed in the study (i.e. H_37_Rv CFU reduction, lung histopathology and delayed time-to-death). While these results are encouraging, we had hypothesized that our rBCG would be effective at inducing an immune reaction that would target latent bacilli. Examination of lung and spleen tissues at chronic phases of infection did not reveal significant additional reductions in CFU or pathology by rBCG. Although mice receiving rBCG were significantly protected from death through the chronic phase of infection compared to naïve mice, mortality rates were not significantly different in mice that received control BCG.

Since it has been shown that *Mtb* possesses at least two active L,D-transpeptidases that catalyze the formation of non-classical linkages during the stationary phase of growth [17], [18], there is the possibility that overproduction of *Rv2518c* alone may be ineffective at creating a strain which contains a sufficient amount of altered peptidoglycan to induce protection against latent *Mtb*. In order to address this possibility, we subjected rBCG to nutrient starvation *in vitro*, and the resulting immunogen, rBCG-NS, was able to protect mice as well as control BCG in all measures of protection assessed. As observed in rBCG, however, we saw no signs that rBCG-NS was able to protect mice better than control rBCG or control BCG against populations of bacilli present during the chronic phase of infection. However, when we examined the cytokine profile elicited by rBCG-NS, we observed increases in lung IFN-γ, and TNF-α before challenge and higher levels of IL-12 after challenge which indicates that rBCG-NS was able to elicit a stronger immune response compared to rBCG. While not always indicative of resulting protection [39], quantitative measurements of cytokines is widely used to assess the ability of a vaccine to induce an immune response.

Results from this study support using *in vitro* nutrient starvation of a live BCG for a wide array of experimental vaccine studies in order to increase Th1-mediated immunological effects. Experimental vaccines using live recombinant BCG to express parasitic or other key non-mycobacterial antigens which may preferentially promote a T-helper type 2 (Th2) cell mediated response could benefit from *in vitro* nutrient starvation to induce a more preferable balance between Th1 and Th2 responses to help alleviate unfavorable Th2 induced asthmatic responses [40]. Likewise, a BCG that induces a stronger Th1 response could potentially increase the inherently positive anti-allergen effects of currently available BCG vaccines [41]. Additionally, studies that involve coadministration of a live rBCG and an adjuvanted subunit vaccine [42] could benefit by increasing the Th1:Th2 balance of the cell mediated immune response potentially offering greater flexibility in choosing an effective adjuvant, often a difficult topic for vaccinologists due to several factors [20]. Finally, one shortcoming of live BCG vaccines is that they have been shown to fail to protect individuals against TB in regions that contain a high burden of TB disease [43] potentially due to the increased presence of environmental mycobacteria in these countries [44]. A BCG vaccine that is able to induce a more robust cell mediated response characterized by higher induction of IFN-γ and IL-12 could potentially alleviate the “blocking” hypothesis presented in [44], especially in regions that administer BCG after potential exposure to mycobacteria through the water or soil can occur (e.g. after a newborn has left the hospital). It is clear from this study that further experiments are needed to determine whether increased host induction of Th1 cytokine resulting from *in vitro* nutrient starvation of rBCG is due to the overexpression of the Ldt_Mt2_, or whether this effect can be recapitulated in wild type BCG.

Although data from experiments using the exponentially grown rBCG do not necessarily support moving forward with this strain in more rigorous testing at this time, experiments to further characterize the effects of *in vitro* nutrient starvation on rBCG and the parental wild-type BCG should be pursued. This rBCG-NS, while able to protect mice as well as the relevant controls, and elicited a stronger immune response in mice marked by higher levels of lung IFN-γ, TNF-α, and IL-12 compared to the parent strain that was grown exponentially. A more robust response in these cytokines offer researchers a useful tool to incorporate customized Th1 responses in studies using live BCG to express heterologous antigens, in studies involving use of adjuvants, and could possibly even help increase resulting protection in BCG immunized populations that do not respond effectively to current BCG.

## Materials and Methods

### Ethics statement

No ethics statement is required for this study. All animal protocols used in this study have been reviewed and approved by the Johns Hopkins Institutional Animal Care and Use Committee (protocol number MO09M101).

### Bacterial strains and *in vitro* growth conditions

*M. bovis* BCG Copenhagen (Danish strain 1331) was purchased from ATCC (Manassas, VA). H_37_Rv was used as the virulent *Mtb* strain for challenge studies. Mycobacterial cultures were grown in Middlebrook 7H9 broth (Difco) supplemented with 0.5% glycerol, 10 µg/mL cycloheximide (Sigma), 0.05% Tween-80 (Sigma), and 10% BBL Middlebrook OADC enrichment (Beckton Dickinson). For colony forming unit (CFU) determination, bacilli were grown on Middlebrook 7H10 agar (Difco) supplemented with 0.5% Glycerol, 10 µg/mL cycloheximide (Sigma), 0.05% Tween-80 (Sigma), and 10% BBL Middlebrook OADC enrichment (Beckton Dickinson). OmniMAX 2 -T1^R^ *E. coli* (Invitrogen) was used in all DNA cloning procedures and was grown according to the manufacturer's directions.

### Plasmid and strain construction

Reagents for DNA cloning procedures were used according to the manufacturers' supplied protocols or as described [45]. The 1,227 base pair *ldt_Mt2_* was cloned from *Mtb* genomic DNA (obtained through the TB Vaccine Testing and Research Materials Contract, Colorado State University) via polymerase chain reaction with primers GW-Rv2518c@1-SP (5′- GGGGACAAGTTTGTACAAAAAAGCAGGCTAGGAGGGAAGGCATGCCAAAGGTGGGGATTG -3′) and GW-Rv2518c@1227-ASP (5′- GGGGACCACTTTGTACAAGAAAGCTGGGTTTACGCCTTGGCGTTA CCG G -3′). The resulting amplicon was purified and recombined into the Gateway entry vector pDONR/Zeo (Invitrogen). The *ldt_Mt2_* coding sequence was fully sequenced (Genewiz), compared to the published sequence in GenBank (http://www.ncbi.nlm.nih.gov/Genbank) and a verified clone with the correct sequence was transferred into the mycobacterial – *E. coli* Gateway shuttle vector pGS400H [46]. The resulting plasmid was electroporated into BCG using standard techniques [47]. Selection of transformants was performed on Middlebrook 7H10 agar in the presence of 50 µg/mL hygromycin at 37°C for 3–4 weeks. Candidates were subcultured and verified using Southern analysis and multiple vials of positive clones were frozen in 15% glycerol at −80°C.

### Measurement of antigen expression

Approximately 1 µg of total RNA isolated from an *in vitro* culture of the recombinant strain at the late exponential phase of growth (A_600 nm_ = 1.2) was treated with TURBO DNase (Ambion) and reverse transcribed into cDNA with random hexamers using the SuperScript First-Strand Synthesis System for RT-PCR (Invitrogen) using the manufacturer's supplied protocol. Real-time detection of *ldt_Mt2_* transcripts was performed using iQ SYBR-Green Supermix (Bio-Rad) with the primers Rv2518c-RT1 (AGCACATCATCATGGACTCG), and Rv2518c-RT2 (CGCTTGACATGGTCGTAGAA) in an iCycler iQ Real-Time PCR Detection System (Bio-Rad). Transcript levels of *ldt_Mt2_* (BCG_2539c) in the parent and the recombinant strain were normalized to *sigA* and fold change in expression level was calculated using the comparative C_t_ method [48].

### Experimental animals

C57/BL6 mice (Charles River Laboratories) were housed in the pathogen-free biosafety level 3 vivarium facility at Johns Hopkins University. Mice were provided food and water *ad libitum* as well as appropriate monitoring and clinical care. All protocols used in this study have been reviewed and approved by the Johns Hopkins Institutional Animal Care and Use Committee (protocol number MO09M101).

### Preparation of immunizing agents and immunizations

Table S1 lists the immunogens used in this study. Twenty eight mice per group were immunized in the lateral tail vein by inoculating 0.2 ml of a suspension containing approximately 1×10^7^ CFU of either control BCG, the recombinant strain (rBCG) or rBCG that was nutrient starved (rBCG-NS). The negative control group of 20 mice did not receive any immunization. BCG and rBCG preparations were made by growing respective strains in 7H9 broth to log phase and diluting to a density of ∼5×10^7^ CFU/mL using phosphate buffered saline (PBS). To prepare rBCG-NS, we grew rBCG in 7H9 broth to log phase, washed twice with PBS containing 0.05% Tween-80 (PBST) then diluted into PBST to a concentration of approximately 5×10^7^ CFU/mL and incubated at 37°C as a standing culture for 1 week. Diluted samples of all suspensions used for immunization were plated onto 7H10 agar to enumerate viable bacilli. At 1 day and 2, 4, and 6 weeks post immunization, the lungs and spleens of vaccinated animals were removed, homogenized and plated onto 7H11 Selective agar in order to assess the *in vivo* growth of the immunizing strains prior to challenge.

### Challenge with virulent *M. tuberculosis*

All mice were challenged in a Glas-col Middlebrook Inhalation Exposure System (Glas-col Inc.) with a suspension of *Mtb* H_37_Rv at 6 weeks following immunization. A log phase culture of H_37_Rv was used to implant ∼3 log_10_ bacilli in the lungs of mice for assessment of growth of the challenge strain. A separate infection was carried out to implant ∼4 log_10_ CFU of H_37_Rv: this is a high dose that results in death of naïve mice and is therefore suitable for measuring protection against virulent *Mtb.* For this assessment, 10 mice from each group that received immunization and 13 mice for the naïve group were used. Diluted samples of all suspensions used for aerosol infection were plated onto 7H10 agar to determine infective dose. Implantation rates were determined by plating lung homogenates from naïve mice obtained one day after aerosol challenge onto 7H11 Selective agar plates.

### Determination of bacterial burden and histopathology

Lungs and spleens of 3 mice per time point were aseptically removed and transferred to a 2 ml screw cap tube containing 2 mm glass beads and homogenized using a Biospec Mini Bead Beater (BioSpec Products) in a total volume of 1 mL of PBS. In order to measure the bacterial burden of the challenge strain in immunized animals, diluted organ homogenates were plated onto Middlebrook 7H11 Selective plates containing 2 µg/mL 2-thiophenecarboxylic acid (Sigma). Total CFU counts were determined following 3–4 weeks of incubation at 37°C. For histopathological studies, lung tissue samples were obtained from mice at 2, 8, and 16 weeks post challenge, fixed in formalin, paraffin imbedded, sectioned and stained with hematoxylin and eosin. Slides were examined using a Nikon Eclipse E8000 microscope outfitted with a Nikon DXM1200 camera and Nikon ACT-1 image acquisition software.

### Cytokine measurements

Lung homogenates in a total volume of 1 mL from sacrificed animals at predetermined times after immunization or subsequent challenge were thawed, diluted 5-fold in PBS, filtered using a 0.22 um syringe filter (Millipore) and assessed using a Bio-Plex Express Mouse group 1 7-plex kit (Bio-Rad). Assay detection was performed using a Bio-Plex 200 System (Bio-Rad) and total lung levels of IL-1β, IL-10, IL-12 (p40), IFN-g, MIP-1a, RANTES, and TNF-a were measured.

### Statistical analysis

The two-tailed Student's t-test was performed to determine significance in CFU measurements. Log-rank test analysis was used to compare survival data using PRISM (Graphpad Software). P values ≤0.05 were used to indicate significant differences.

## Supporting Information

Table S1 List and description of immunogens used in this study.
(0.03 MB DOC)

Figure S1 Plasmid map of pGS400H-2518. The E. coli/Mtb shuttle plasmid pGS400H is a Gateway-enabled derivative of pMV361 which accepts fragments from Gateway entry plasmids and features expression of the exogenous gene from the constitutively strong hsp60 promoter region of mycobacteria. This plasmid integrates stably into the mycobacterial genome at the attB locus using the plasmid-encoded mycobacteriophage L5 integrase, and features hygromycin resistance for selection of transformants.
(1.19 MB TIF)

Figure S2 Southern blot data used to verify the correct genotype of rBCG transformants. (A) Genomic DNA from the parental BCG strain (Lane 2) and putative rBCG transformants (Lanes 3–12) were digested with XhoI, run on a 0.8% agarose gel transferred to a nylon membrane and hybridized to a DIG-labelled probe homologous to a fragment of Mtb gene Rv2518c. Lane 1 contains commercial Dig labeled molecular weight markers and the fragment sizes are denoted. The expected fragment size for the parental strain, 3 kilobases was observed in lane 2. Lanes 11 and 12, among others, display the correct fragment sizes for rBCG which is 3 kilobases (the wild-type copy) and 10 kilobases (the exogenous copy). (B) The nylon membrane from (A) was stripped using the manufacturers supplied protocol and hybridized to a DIG-labeled probe homologous to a fragment of the Hygromycin resistance gene present on pGS400H-2518. Lane 1 contains commercial Dig labeled molecular weight markers and the fragment sizes are denoted. There should be no fragment expected for the parental strain, as was observed in lane 2. Lanes 11 and 12, display the correct fragment sizes for rBCG which is 10 kilobases.
(5.39 MB TIF)
