# Supplementary material for: Protective Efficacy of BCG Overexpressing an L,D-Transpeptidase against M. tuberculosis Infection
Source: PLoS One. 2010 Oct 29;5(10):e13773. doi: 10.1371/journal.pone.0013773 (PMC2966435; doi:10.1371/journal.pone.0013773)
Supplement: Table S1 — List and description of immunogens used in this study. (0.03 MB DOC) [file pone.0013773.s001.doc]

| **Immunogen** | **Source** | **Description** |
| --- | --- | --- |
| BCG | *in vitro* culture | *M. bovis* BCG Copenhagen |
| rBCG | *in vitro* culture | BCG overproducing ldt_Mt2_ |
| rBCG-NS | *in vitro* culture | BCG overproducing ldt_Mt2_ that has been nutrient starved *in vitro* |
