# Supplementary figures and images for: Protective Efficacy of BCG Overexpressing an L,D-Transpeptidase against M. tuberculosis Infection
Source: PLoS One. 2010 Oct 29;5(10):e13773. doi: 10.1371/journal.pone.0013773 (PMC2966435; doi:10.1371/journal.pone.0013773)

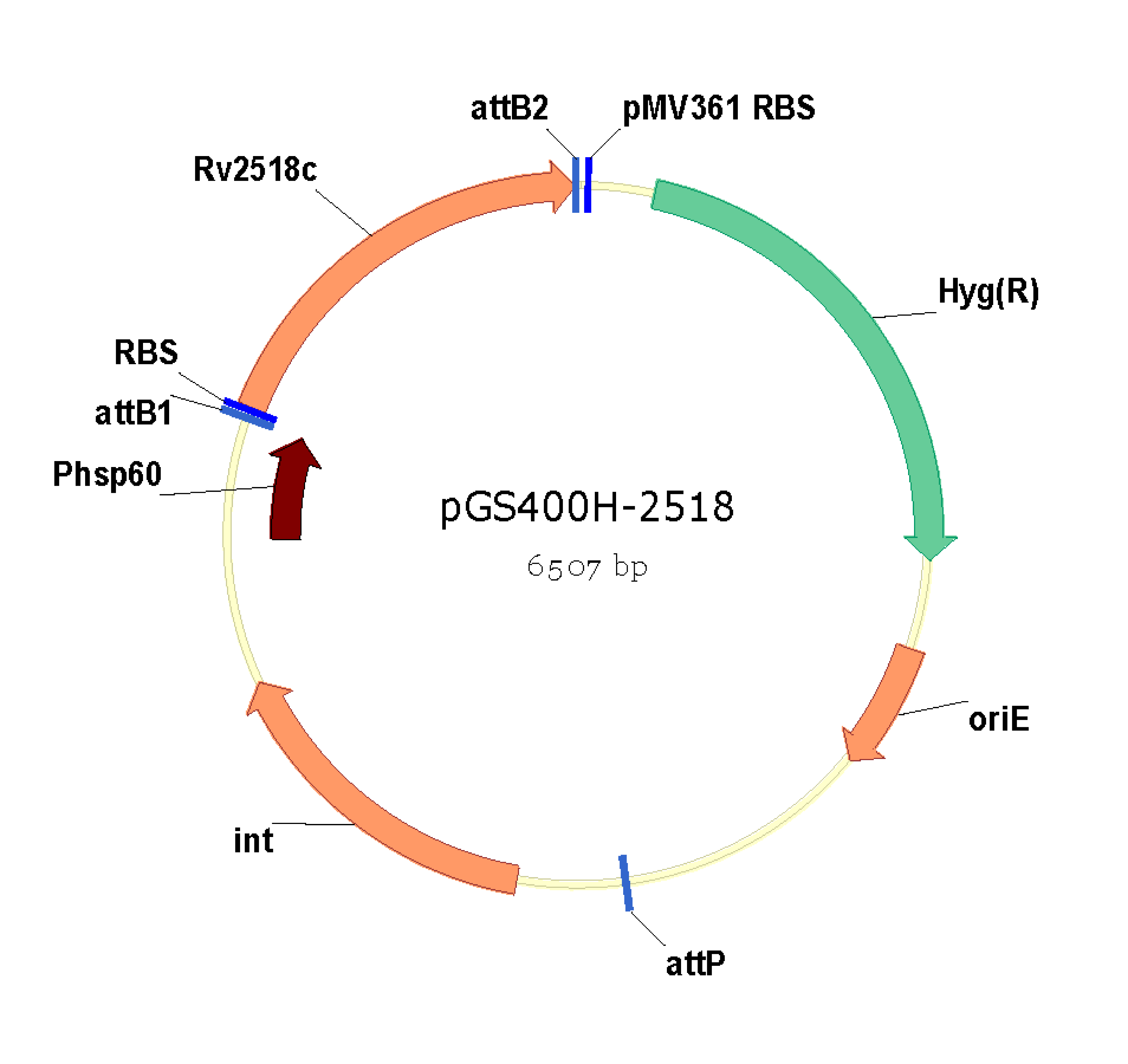

Supplement: Figure S1 — Plasmid map of pGS400H-2518. The E. coli/Mtb shuttle plasmid pGS400H is a Gateway-enabled derivative of pMV361 which accepts fragments from Gateway entry plasmids and features expression of the exogenous gene from the constitutively strong hsp60 promoter region of mycobacteria. This plasmid integrates stably into the mycobacterial genome at the attB locus using the plasmid-encoded mycobacteriophage L5 integrase, and features hygromycin resistance for selection of transformants. (1.19 MB TIF) [file pone.0013773.s002.tif]

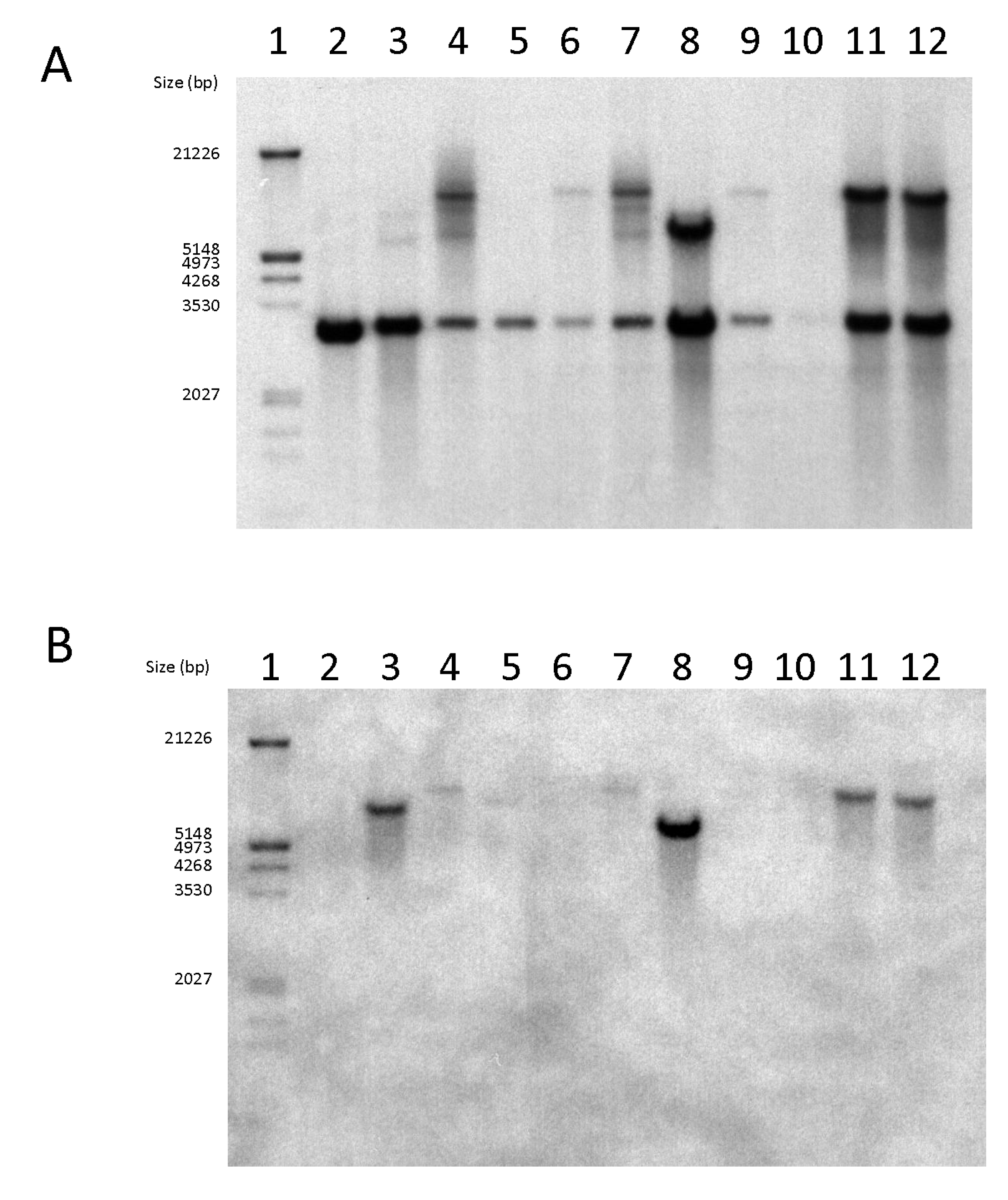

Supplement: Figure S2 — Southern blot data used to verify the correct genotype of rBCG transformants. (A) Genomic DNA from the parental BCG strain (Lane 2) and putative rBCG transformants (Lanes 3–12) were digested with XhoI, run on a 0.8% agarose gel transferred to a nylon membrane and hybridized to a DIG-labelled probe homologous to a fragment of Mtb gene Rv2518c. Lane 1 contains commercial Dig labeled molecular weight markers and the fragment sizes are denoted. The expected fragment size for the parental strain, 3 kilobases was observed in lane 2. Lanes 11 and 12, among others, display the correct fragment sizes for rBCG which is 3 kilobases (the wild-type copy) and 10 kilobases (the exogenous copy). (B) The nylon membrane from (A) was stripped using the manufacturers supplied protocol and hybridized to a DIG-labeled probe homologous to a fragment of the Hygromycin resistance gene present on pGS400H-2518. Lane 1 contains commercial Dig labeled molecular weight markers and the fragment sizes are denoted. There should be no fragment expected for the parental strain, as was observed in lane 2. Lanes 11 and 12, display the correct fragment sizes for rBCG which is 10 kilobases. (5.39 MB TIF) [file pone.0013773.s003.tif]
